# Graphene/GaSe-Nanosheet Hybrid: Towards High Gain and Fast Photoresponse

**DOI:** 10.1038/srep19161

**Published:** 2016-01-18

**Authors:** Rongtao Lu, Jianwei Liu, Hongfu Luo, Viktor Chikan, Judy Z. Wu

**Affiliations:** 1 Department of Physics & Astronofmy, University of Kansas, Lawrence, KS 66045, USA; 2 Department of Chemistry, Kansas State University, Manhattan, KS 66506.

## Abstract

While high photoconductive gain has been recently achieved in graphene-based hybrid phototransistors using semiconductor two-dimensional transition/post-transition metal dichalcogenides or quantum dots sensitizers, obtaining fast photoresponse simutaneously remains a challenge that must be addressed for practical applications. In this paper we report a graphene/GaSe nanosheets hybrid photodetector, in which GaSe nanosheets provide a favorable geometric link to graphene conductive layer through van Der Waals force. After a vacuum annealing process, a high gain in exceeding 10^7^ has been obtained simitaneously with a dynamic response time of around 10 ms for both light on and off. We attribute the high performance to the elimination of possible deep charge traps, most probably at the graphene/GaSe nanosheets interface. This result demonstrates high photoconductive gain and fast photoresponse can be achieved simultaneously and a clean interface is the key to the high performance of these hybrid devices.

Graphene has attracted extensive attention due to its unique electronic structure^1^, two dimensional nature, ultrahigh mobility (up to 4 × 10^4^ cm^−2^V^−1^s^−1^ for unsuspended graphene at room temperature)^2^,^3^, flexibility and chemical stability. Graphene optoelectronics has emerged as an important area, especially graphene photodetectors have been approved for high frequency detection up to 40 GHz^4^,^5^,^6^. However, the device performance, especially the responsivity, has been limited by the low optical absorption of graphene^7^ and lack of high gain mechanism in the device configuration. This has prompted intensive efforts recently in implementing various nanostructures to improve photoabsorption in ultraviolet to near infrared range^8^,^9^,^10^,^11^,^12^ and exciting progress has been made in raising responsivity to around 10 mA/W by applying plasmonic metal nanoparticles^10^,^11^, and further to 350 mA/W by incorporating efficient ionic liquid gating configuration^12^. Recently, responsivity was further enhanced to several A/W by engineering graphene band structures^13^, stacking graphene as vertical p-n junctions^14^, or integrating energy filtering barrier into graphene^15^. In particular, an ultrahigh photoconductive gain of 10^8^ charge carriers per photon and hence a high responsivity up to 10^7^ A/W were obtained in a hybrid graphene/semiconductor quantum dot (QD)^16^, graphene/two-dimensional transition/post-transition metal dichalcogenides (TMDC), such as MoS_2_ and WSe_2_^17^,^18^,^19^, field-effect devices (FETs). Recent progress relevant to graphene/QD and graphene/TMDC hybrid photodetectors is compared in Table 1.

In these devices, one type of the photoexcited carriers in the PbS-QD (or TMDC nanosheets) absorption layer is transferred into the graphene layer and circulate many times due to the high mobility of graphene within the lifetime of the oppositely charged carriers in the PbS-QDs, resulting in a photoconductive gain. The oppositely charged carriers are trapped in the QD layer within their lifetime, providing a photo-gating effect on the PbS-QD/graphene FET (GFET) channel conductance^16^,^20^,^21^. Theoretically, under equilibrium excitation, the photocurrent *I*_ph_ in such a device is proportional to the photoconductive gain *g*^22^


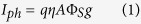


where *q* is electron charge, *η* is quantum efficiency, *A* is detector area, *Φ*_*s*_ is incident light photon flux density^22^. The gain can be described as 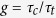, where 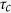 is the life time of excess carriers in the absorber layer and directly determines the transient dynamic response time of the observed photoresponse, and 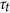 is the carrier transit time in the photoconductive channel between electrodes^22^. The transit time 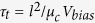 is proportional to the square of channel length *l* and inversely proportional to the carrier mobility in photoconductive channel *μ*_c_ and the bias voltage *V*_bias_^22^. Thus the high mobility in graphene is claimed a key factor facilitating the high gain up to 10^8^ reported in graphene/PbS-QD and graphene/TMDC nanosheets hybrid photodetectors^16^,^17^,^18^,^19^.

While these results are exciting, restrictions exist in slow transient response decay time in these hybrid photodetectors with photoresponse time typically in exceeding 100 ms due to the surface/interface charge trap states^16^,^23^. While the existence of massive deep trap states facilitates the high gain, they are detrimental to the photoresponse speed and consequently hinder practical photodetection applications. Addressing this issue is crucial. In this work, graphene/GaSe nanosheet FETs were selected considering the similar layered structures of the graphene and GaSe could form an efficient van Der Waals interface, allowing elimination of the cumbersome ligand exchange processes. Gallium selenide (GaSe), a III-VI post-transition metal chalcogenide with layered structure as shown in Fig. 1(a), is an alternative candidate with a direct band gap of about 2.11 eV and a 25 meV smaller indirect band gap^24^,^25^. Especially, GaSe is chemically stable in air^26^ and has a thermal stability at temperature up to 600 ^o^C^27^ (see supplementary information for more details of GaSe). Exfoliated few-layer GaSe nanosheets were recently synthesized for photodetection, and a responsivity of 2.8 A/W upon 254 nm laser illumination was reported with the response time in a wide range from 20 ms to a few seconds^27^. GaSe nanosheet with larger layer number was later reported with a responsivity of 17 mA/W to 405 nm laser illumination^28^, similar to the epitaxial GaSe films grown on flexible mica^29^. In addition to these explorations on individual GaSe sheets, GaSe networks with sub-*μ*m elemental dimension were reported with a peak responsivity of 1.4 A/W under 240 nm illumination^30^. The layered structure and the band gap tunability via layer numbers in GaSe nanosheets make them excellent photosensitizers on graphene to form a broad-band graphene/nanosheet hybrid for photodetection. In particular, elimination of ligands can simplify the device structure and allow a pathway to achieve high gain and fast photoresponse by reducing excessive trap states associated to the ligands. The graphene/GaSe-nanosheet hybrid therefore combines the superior properties of high charge mobility in graphene for fast transit of the transferred charges and efficient quantum confinement of the opposite charges on the nanosheets^31^,^32^,^33^. A vacuum annealing process^34^ was applied in this work for interface cleaning. The clean graphene/GaSe nanosheet interface was found not only to enable fast photoresponse by reducing excessive trap states, but also facilitate the charge carrier injection from photosensitizer layer to the electrode, which are the key to the first achievement of both the high gain of 10^7^–10^8^ and the fast photoresponse to visible light with both light-on and light-off time constants as short as 10 ms in the graphene/GaSe-nanosheet FET phototransistors.

## Results and Discussions

**Figure 1:**
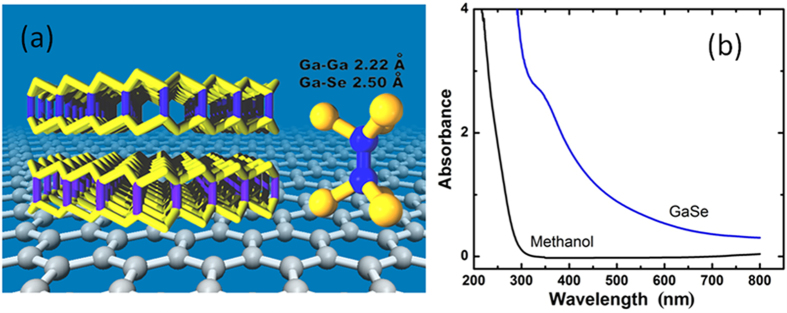
(**a**) Molecular structure of GaSe and the diagram of the graphene/GaSe-nanosheet hybrid; (**b**) Optical absorbance spectrum of GaSe nanosheet solution.

GaSe nanosheets were synthesized from bulk GaSe and details are summarized in Methods^35^. Figure 1(b) shows the optical absorbance of GaSe-nanosheets solution in methanol in the wavelength range of 300–800 nm, where the control methanol shows comparatively flat absorbance profile with no clear feature in such a range. The absorbance of GaSe-nanosheets solution increase monotonically with decreasing illumination wavelength and the increase slope improves from about 600 nm, which is slightly blue shifted from the bulk value of the band edge expected for GaSe nanosheets^36^. The lack of well defined absorption edge is due to variable layer numbers of 1–10 and lateral size of the nanosheets ranging from few to few tens of nanometers as evidenced in TEM analysis [see Supplementary Information, Figure S1].

**Figure 2:**
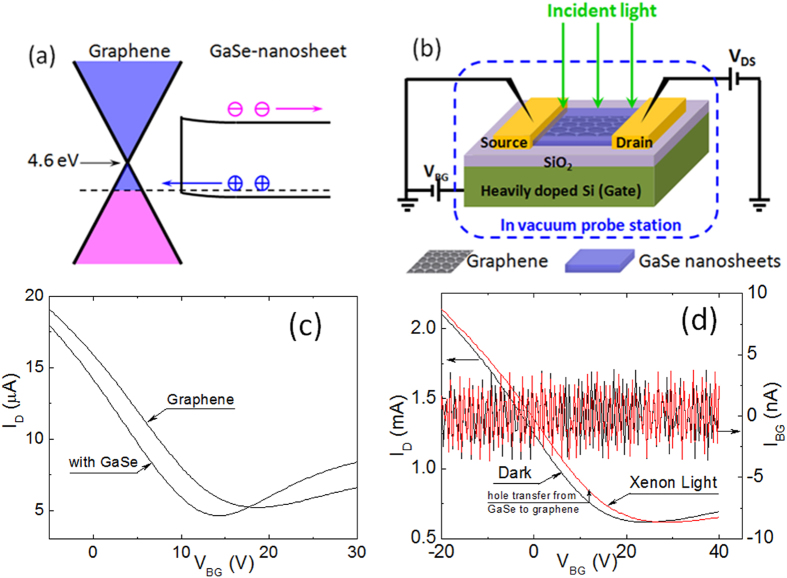
(**a**) Schematic graphene/GaSe-nanosheet interface band diagram; (**b**) Illustration of the graphene/GaSe-nanosheet hybrid GFET and measurement setup; (**c**) *I*_D_ vs. *V*_BG_ curves of GFET with/without GaSe nanosheets in dark, *V*_SD_ = 10 mV. (**d**) GFET transport property of *I*_D_ vs. *V*_BG_ and *I*_BG_ vs. *V*_BG_ curves in dark and under 155 mW/cm^2^ Xenon light illumination; *V*_SD_ = 1 V.

The graphene/GaSe-nanosheet interface band diagram is illustrated schematically in Fig. 2(a). The photogenerated holes transfer into graphene, leading to further p-doping in graphene layer, and the electrons are trapped in the nanosheet layer due to the quantum confinement. The device configuration and measurement setup diagram is illustrated in Fig. 2(b). In order to fabricate GFETs, CVD graphene transferred on SiO_2_ (90 nm)/Si substrates was patterned into micro-channels and Au/Ti electrodes were fabricated on top. The GaSe-nanosheet/methanol solution was deposited onto the graphene channels to form the graphene/GaSe nanosheet hybrid photodetector. The source-drain current (*I*_D_) versus back gate voltage (*V*_BG_) curves with/without GaSe nanosheets in dark are compared in Fig. 2(c). After applying GaSe, the Dirac point shifts to the left for a few volts, together with a slight decrease in minimum conductivity. These changes became negligible after vacuum annealing shown in Fig. 2(d), suggesting the mechanism responsible for the the Dirac point shift and decrease of minimum conductivity decrease may be associated to the solvant not fully removed before the vacuum annealing performed prior to the optoelectronic measurements (see Methods for more details). The *I*_D_ versus *V*_BG_ in dark and under 155 mW/cm^2^ Xenon light illumination taken on a representative graphene/GaSe-nanosheet hybrid GFET is shown in Fig. 2(d). The *V*_SD_ (or *V*_bias_ in this paper) was kept at a larger bias of 1 V, which is needed to obtain higher photocurrent as described in the later part of this paper. Under an incident light, the Diract point shifts further to the right side as anticipated from photogenerated hole doping, inducing a positive photocurrent response at zero gate voltage bias as a result of the photo-gating effect, which is qualitatively consistent with previously reported graphene/PbS-QD phototransistors^16^. Gate leakage current *I*_BG_ vs. *V*_BG_ curves are also shown in Fig. 2(d), together with low leakage currents of only few nA observed in all devices in this work.

**Figure 3:**
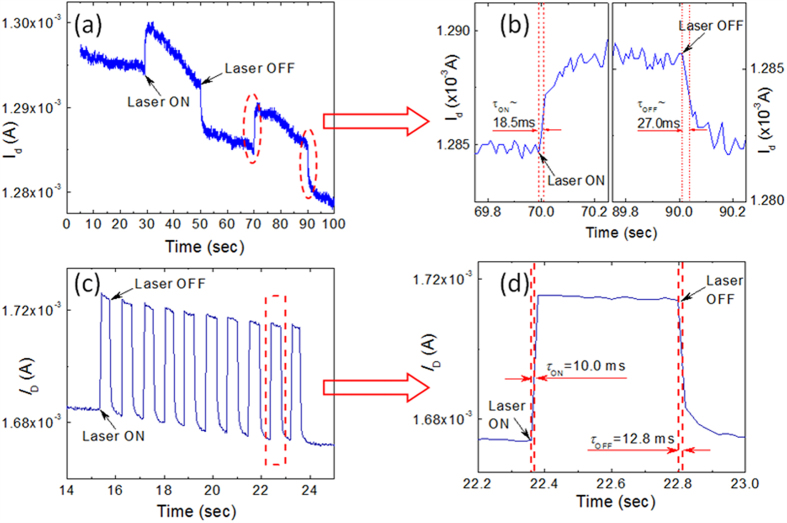
Dynamic response *I*_D_ vs. time curves of graphene/GaSe-nanosheet hybrid GFET upon 532 nm incident laser ON/OFF modulation. (**a**,**b**) before vacuum annealing, incident laser intensity ~188 mW/cm^2^. (**c,d**) after vacuum annealing, incident laser intensity ~198 mW/cm^2^. (**a**,**c**) are full dynamic response spectra, (**b**,**d**) shows zoom-in view of rise/decay. Response time was estimated using magnitude change criteria. Back gate voltage *V*_BG_ = 0 V, and source-drain voltage *V*_SD_ = 1 V for all measurements.

Figure 3 compares the dynamic phtoresponses measured on a graphene/GaSe-nanosheet hybrid GFET before [Fig. 3(a,b), ~188 mW/cm^2^] and after [Fig. 3(c,d), ~198 mW/cm^2^] the vacuum annealing to a 532 nm laser at comparable incident power intensities, and the back gate voltage *V*_bg_ = 0 V and source-drain voltage *V*_d_ = 1 V were maintained for both measurements. While the two curves follow a similar trend qualitatively, they differ considerably in both the magnitide of the photoresponse, defined as the difference of the source-drain current *I*_D_ under illunimation with respect to in dark (or photocurrent Δ*I*_D_), and the photoresponse time constants, defined as the consumed time corresponding to 50% magnitude rise/decay change upon incident light on/off. After the vacuum annealing, the Δ*I*_D_ increased by a factor of eight, which illustrates the importance in achieving a clean GFET channel interface since polar molecules adsorbed the channel respond to the electric field applied to the GFET and reduce the effective gating field sensed by GFET^34^. Since the effect of the vacuum annealing is permanent, the removed polar molecules are probably those from chemicals employed in graphene transfer and GFET device fabrication, instead of air molecules. The response times of 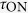 (laser on) and 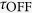 (laser off) are 18.5 ms and 27.0 ms, separately, before the annealing, and are greatly improved by more than 50% after annealing. After vacuum annealing, by further expanding the dynamic response upon one laser pulse, as shown in Fig. 3(d), the response times of 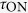 ~ 10.0 ms and 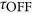 ~ 12.8 ms (i.e. the life time 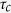
~10 ms) upon the laser ON/OFF switches, respectively, can be retrived. Compared to several other recent reports on graphene-based hybrid photodetectors (Table 1), while the response rise time 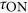 is comparable to the lower end of 10–20 ms reported in exfoliated graphene/PbS-QD phototransistor^16^, it is more than one order of magnitude smaller than that observed in its counterpart using CVD graphene/PbS-QD^20^. In particular, the fast decay time 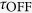 obtained in this work on graphene/GaSe-nanosheet GFET represents a remarkable improvement over the 100 ms-1 second reported in the exfoliated graphene/PbS-QD phototransistor^16^ and a few seconds in its CVD graphene counterpart^20^. Furthermore, the comparable 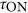 and 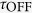 result in the achievement of a symmetric dynamic photoresponse in the graphene/GaSe-nanosheet hybrid GFET. This result suggests the additional deep charge traps associated to the ligants and graphene/nanosheet interface defects may be the primary mechanisms that prevent achievement of the symmetric and fast dynamic photoresponse in the previously reported graphene/nanosheet hybrid photodetectors. Removing or minimizing these deep charge traps will be a key towards meeting the transient dynamic response compatible to practical imaging applications without the extra optimizations for further processing of the sensitive ligand exchange^23^ and applying additional back-gate reset protocol^16^.

It should be noted that this fast photoresponse is obtained together with a high gain in exceeding 10^7^. For example, at *V*_BG_ = 0 V and incident power intensity of 198 mW/cm^2^, the carrier transient time 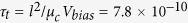
second, which is estimated from *l* = 5 *μ*m, *μ*_c_ ~ 320 cm^2^V^−1^s^−1^ [extracted from Fig. 2(c)], and *V*_bias_ = 1 V, and 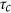 ~ 10 ms lead to a high gain of 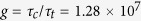. In fact, a higher gain of 10^8^ is obtained at lower incident power intensity of 5.7 × 10^−3^ mW/cm^2^ in our measurements. This result therefore demonstrates the feasibility of simultaneous achivement of both the high gain and fast response in the graphene/GaSe-nanosheet hybrid GFET photodetectors.

**Figure 4:**
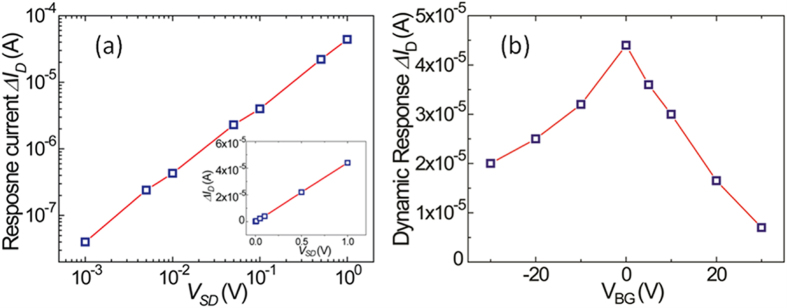
(**a**) Photoresponse current Δ*I*_D_ as function of applied source-drain voltage *V*_*SD*_ at *V*_BG_ = 0 V. The linear dependence confirms the photoconductor property. Inset shows the curve in linear scales. *V*_BG_ = 0 V. (**b**) Δ*I*_D_ as function of back gate voltage *V*_BG_ at *V*_SD_ = 1 V. Laser intensity was kept at 198 mW/cm^2^ for both measurements.

The photocurrent Δ*I*_D_ as function of source-drain bias voltage *V*_SD_ (log-log scales) is summarized in Fig. 4(a) and its inset shows the same curve in linear scales at the back gate voltage *V*_BG_ of 0 V and laser intensity of 198 mW/cm^2^. Apparently, the photocurrent Δ*I*_D_ is linearly dependent on the bias *V*_SD_ in the entire measurement range, as described in equation (1), strongly supporting the photoconductive response mehanism in this graphene/GaSe-nanosheet hybrid phototransistor^22^. Figure 4(b) shows the correlation of Δ*I*_D_ vs. *V*_BG_ at a constant *V*_SD_ = 1 V. Interestingly, the maximum photocurrent was observed at ~*V*_BG_ = 0 V while it decrases monotonically with increasing *V*_BG_ abosulte value, which is qualitatively consistent with the photoresponse observed under Xenon light, as shown in Fig. 2(c).

**Figure 5:**
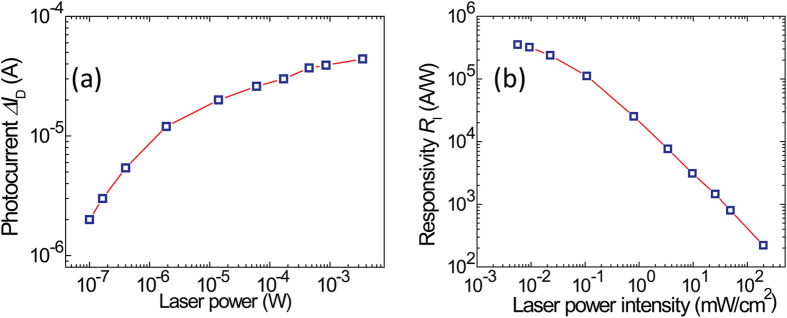
(**a**) Photocurrent Δ*I*_D_ vs. laser power (in log-log scales) at *V*_SD_ = 1 V and *V*_BG_ = 0 V. (**b**) Photoresponsivity *R*_I_ as function of laser power intensity, calculated from (**a**).

The laser power intensity affects responsivity as summarized in Fig. 5. As shown in Fig. 5(a), the photocurrent increases monotonically with the increasing laser power across four and a half orders of magnitudes. Further calculation of the photoresponsivity *R*_I_ at different laser intensities is summarized in Fig. 5(b), and the trend of the monotonic decreasing *R*_I_ with increasing laser power intensity is in good agreement with ideal photoconductive response^22^ and is consistent to that on graphene/PbS-QD detectors^16^,^17^. The maximum *R*_I_ ~ 3.5 × 10^5^ A/W is obtained at 5.7 × 10^−3^ mW/cm^2^, the minimum controllable laser power intensity in our setup, and higher responsivity value is expected at lower incident laser intensity by following the trend in Fig. 5(b). The 1/f like noise spectrum [see Supplementary Information, Figure S2] has been characterized from this graphene/GaSe-nanosheet phototransistor in the case of keeping *V*_SD_ = 1 V, *V*_BG_ = 0 V and frequencies about 1.6 Hz, giving a detectivity D* up to 1.1 × 10^10^ cm.Hz^1/2^/W at 5.7 × 10^−3^ mW/cm^2^ intensity for 532 nm incident laser, where detectivity is calculated as 
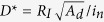
, *A*_d_ is the detection area and 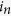 is the noise current^22^. The high responsivity and detectivity are several orders of magnitude greater than those obtained in most previously reported graphene-based photodetectors optimized with plasmonic structures^10^,^11^,^12^. While the *R*_I_ is lower than the best reported value of 10^6^–10^7^ A/W taken on the graphene/PbS-QD and graphene/TMDC hybrid photodetectors, several factors should be considered before a direct comparison is made. First, it is widely reported that the observed photoresponsivity decreases monotonically with increasing incident light power, and all the previously reported higher photoresponsivity results were obtained at light power intensity much lower than that of this work^16^,^18^,^20^. In the case of the graphene/PbS QD hybrid case, for example, *R*_*I*_ decreases linearly in the log-log scale from about 10^6^ A/W at around 10^−5^ mW/cm^2^ to about 20 A/W at around 10 mW/cm^2^ 20. Since the minimum light power intensity used in our experiment is higher by about two-to-six orders of magnitude than those where the highest *R*_I_ results were obtained in hybrid photodetectors^16^,^18^ (in Ref. 16 the maximum responsivity of 5 × 10^7^ A/W and maximum detectivity of 7 × 10^13^ cmHz^1/2^/W were obtained at 8 fW, corresponding to an incident power intensity of 1 × 10^−9^ mW/cm^2^ for the laser spot diameter of 1 mm.), considerably higher *R*_I_ values are expected on graphene/GaSe-nanosheet hybrid GFET photodetectors at lower light intensity. Furthermore, it should be pointed out that the high gain (and high *R*_I_, D*) and fast response time are achieved simultaneously at zero *V*_BG_ in this work on graphene/GaSe-nanosheets. This is in contrast to the mandatory high *V*_BG_ in the range of −60 V to −40 V required to observe photoresponse in graphene/MoS_2_ hybrids^18^. In the graphene/PbS-QD hybrid case^16^, the best *R*_*I*_ was observed at *V*_BG_ ~ −20 V, which is considerably better than the *R*_*I*_ at zero *V*_BG_ by a factor of 3–4. Finally, all the devices studied in this work were made using CVD graphene. In contrast, the high *R*_I_ records obtained in previous graphene hybrid photodetectors were all obtained on exfoliated graphene flakes^16^,^18^. Since CVD graphene is particularly advantageous to practical applications, the demonstration of simultaneous high gain and fast photoresponse on CVD graphene is important.

## Conclusion

In summary, we have developed a graphene/GaSe-nanosheet hybrid GFET for photodetection. High gain in exceeding 10^7^ and fast photoresponse with response time constants around 10 ms have been demostrated simultaneously for the first time to our knowledge. In particular, the demonstrated fast and symmetric dynamic photoresponse is so far the best achieved in the graphene-based hybrid devices integrated with quantum dots or other two dimensional materials. The key to this achievement is the elimination of interfacial deep charge traps. The method developed for fabrication of GaSe nanosheets on CVD graphene is robust and low-cost, which is promising for large-scale device fabrication with compatibility to existing microfabrication procedures and on-chip integration with Si-based readout circuits.

## Methods

### Graphene/GaSe nanosheet FETs fabrication

Single layer graphene fabricated using chemical vapor deposition (CVD) was transferred onto heavily doped Si(100) substrate with 90 nm thermal oxide and annealed in Ar:H_2_ (500 sccm:500 sccm) at 400 ^o^C for 15 min to clean up residues of transfer process^37^,^38^. The graphene FET channels about 20 μm wide and 5 μm long were patterned using multiple steps of lithography and etching, as we reported earlier^12^. Au(88 nm)/Ti(2 nm) source and drain electrodes were deposited in a high vacuum electron-beam evaporator. The GaSe nanosheets were synthesized based on the same procedure reported previously^35^. Briefly, 200 mg of bulk GaSe [Gallium(III) selenide, 99.99% (metals basis) from Alfa Aesar] was added to 50 mL of methanol and sonicated for 24 hours at 42,000 Hz with 35 W power. Following the sonication, the GaSe slur was centrifuged at 8000 rpm for 15 minutes to remove the bulk GaSe from the solution. Only the GaSe nanosheets remain in the supernatant and the GaSe-nanosheet/methanol solution was directly used for further experiments. The graphene/GaSe-nanosheet GFET samples were obtained by casting a droplet of GaSe-nanosheet/methanol solution on the GFET channel. The samples were further cleaned prior to the measurements by keeping samples in high vacuum of <2 × 10^−6^ Torr for about three days in the probe station, which was found critical to removing the polar molecules adsorbed to the GFET channel during the device fabrication (shorter vacuum annealing time of 1 day was found not sufficient^34^) and to achieving the high optoelectronic performance. This cleaning was found effective and permanent and no additional vacuum cleaning was necessary if the samples were exposed to atmosphere after the first vacuum cleaning.

### Material and device characterization

Transmission electron microscopy (TEM) imaging of the GaSe nanosheets was done at room temperature using a FEI Tecnai F20 XT field emission transmission electron microscope. Samples were prepared by directly depositing the sonicated GaSe-nanosheet/methanol solution onto a lacey carbon copper grid. The resolution of the instrument was 0.25 nm in TEM mode and 0.18 nm in scanning transmission electron microscopy mode with high angular annular diffraction (STEM-HAAD) mode for elemental analysis. Energy-dispersive X-ray (EDX) spectroscopy was conducted to analyze the sample’s chemical composition.

### Photoresponse measurement

The back-gated field effect transport properties were characterized using an Agilent B1505A semiconductor device analyzer in a high-vacuum probe station of <2 × 10^−6^ Torr at room temperature. A Xenon lamp was used as broad-band light source for preliminary tests and a 532 nm laser was used for detailed dynamic photoresponse measurements. The laser spot of 1.5 mm in diameter provided a uniform illumination over the GFET channel region. The power intensity was tuned using a continuously variable neutral density filter and calibrated using a Coherent FieldMaxII power meter with OP-2 probe. Electrical current noise spectra across the GFET channel were characterized by measuring the noise fluctuation on a resistor in serial with the GFET using a Stanford Research SR760 spectrum analyzer while keeping the required source-drain voltage across GFET channel.

## Additional Information

**How to cite this article**: Lu, R. *et al.* Graphene/GaSe-Nanosheet Hybrid: Towards High Gain and Fast Photoresponse. *Sci. Rep.* **6**, 19161; doi: 10.1038/srep19161 (2016).

## Supplementary Material

Supplementary Information

## Figures and Tables

**Table 1:** Comparison of recent graphene/QD and graphene/TMDC hybrid photodetectors (room temperature).

Reference	Material	Gain	R_I_ (A/W)	D* (cm.Hz^1/2^/W)	Response time	Light source and min. power
Konstantatos *et al*. 2012. [Ref. 16]	Graphene/PbS-QD	10^8^	5 × 10^7^	7 × 10^13^	*τ*_ON_~10–20 ms *τ*_OFF_~0.1–1 sec	532 nm laser,~10^−9^ mW/cm^2^
Sun *et al*. 2012 [Ref. 20]	Graphene/PbS-QD	N/A	1 × 10^7^	N/A	*τ*_ON_~0.3 sec *τ*_OFF_~1.7 sec	895 nm LED, 14.5 nW/cm^2^
Zhang *et al*. 2012 [Ref. 21]	Graphene/PbS-QD	N/A	1.7 × 10^3^	N/A	not specified, estimated > 10sec	halogen lamp, 200 μW/cm^2^
Roy *et al*. 2013 [Ref. 18]	Graphene/MoS_2_	4 × 10^10^ (Low-T)	5 × 10^8^	N/A	~1 sec	635 nm LED, 1 fW/μm^2^
Zhang *et al*. 2014 [Ref. 19]	Graphene/MoS_2_	10^8^	1.2 × 10^7^	N/A	No dynamic response reported	650 nm laser, 0.01 W/cm^2^
