# Supplementary material for: Graphene/GaSe-Nanosheet Hybrid: Towards High Gain and Fast Photoresponse
Source: Sci Rep. 2016 Jan 18;6:19161. doi: 10.1038/srep19161 (PMC4726015; doi:10.1038/srep19161)
Supplement: Supplementary Information [file srep19161-s1.doc]

Supplementary Information

# **Graphene/GaSe-Nanosheet Hybrid: Towards High Gain and Fast Photoresponse**

*Rongtao Lu^1,*^, Jianwei Liu^1^, Hongfu Luo^2^, Viktor Chikan^2^ and Judy Z. Wu^1,*^*

^1^ Department of Physics & Astronomy, University of Kansas, Lawrence, KS 66045, USA

E-mail: [rt_lu@yahoo.com](mailto:rt_lu@yahoo.com), [jwu@ku.edu](mailto:jwu@ku.edu). ^2^ Department of Chemistry, Kansas State University, Manhattan, KS 66506

**1. Ligand free layered GaSe**

In the bulk form, GaSe has a direct band gap of about 2.11 eV and a 25 meV smaller indirect band gap. When making into nanosheets, the direct band gap was found blue shifted due to the quantum confinement effect and decreases monotonically with the number of GaSe layers in the nanosheets, approaching the bulk value at large layer numbers. There are two different types of excitons present in GaSe. One belongs to the direct transition and the 1S state is 20 meV below the direct band edge transition. This exciton shows **k**_x_, **k**_y_ and **k**_z_ dependence (three dimensional exciton) having a Bohr radius of 31 Ǻ (n=1 direct exciton), which corresponds to approximately five Se-Ga-Ga-Se layers. The size dependence of the second peak of the direct excitonic series has also been determined and found to be 52 Ǻ. The indirect exciton lies below the indirect transition by 30 meV. The energy convergence of the excitonic peaks suggests the two dimensional nature of the exciton. Also, one would expect a highly anisotropic peak series just as it has been proven that this series only allowed for E||c polarization of the incident light.

The primary usage of GaSe is an efficient non-linear crystal for frequency doubling in the middle infrared. This is possible because, the high damage threshold and conversion efficiency of the crystal (up to 36%). GaSe nanosheets and nanorods have been also fabricated and the quantum confinement effects on electrical and optical properties have been investigated.

**2. TEM images of GaSe nanosheets**

**Figure S1 (a)** and **(b)** show the TEM images of GaSe nanosheets drop-casted from GaSe-nanosheet/methanol solution. The presence of both gallium and selenium is confirmed from the EDX measurements as shown in **Figure S1(c)**. From the TEM images we can identify two different structures, the thin sheets of exfoliated GaSe and the spherical structures consisting of smaller aggregates, which was probably due to the aggregation of smaller GaSe layers as methanol was evaporating after sample deposition to the TEM grid. Previously, studies on GaSe nanosheets also indicated similar tendency of aggregation of the materials. The main driving force of the aggregation is van der Waals interaction that is also responsible to keeping the GaSe sheets together in the bulk form. Closer look at some of these spherical aggregates reveal that some of them are hollow inside, as shown in **Figure S1(a)**. The GaSe sheets can be seen on the TEM images [**Figure S1(b)**], which also exhibit similar ‘wrinkling’ behavior to the single sheet graphene. The majority of the GaSe is observed with a dimension of few to few tens of nm, and is believed to be exhibiting strong quantum confinement effects, which is evidenced by the absorption spectrum in **Figure 1(b)** showing blue shift compared to the bulk GaSe’s bandgap.


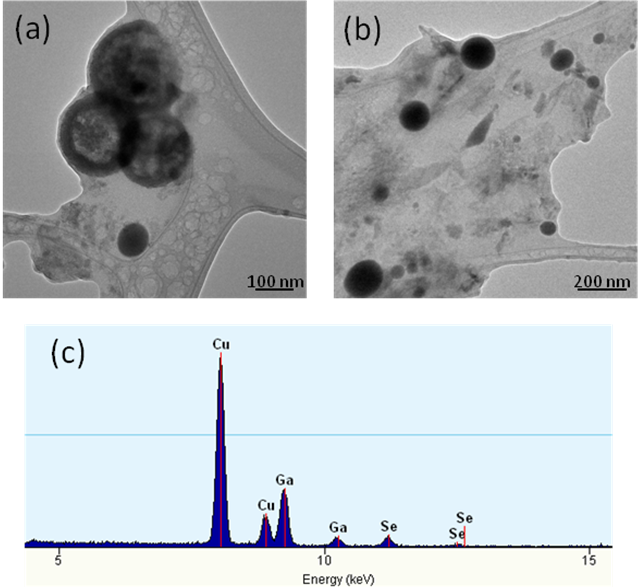


**Figure S1:** TEM images [(a) and (b)] and EDX [(c)] of GaSe nanosheets.

**3. Noise spectrum of graphene/GaSe-nanosheet phototransistor**

The noise spectrum [**Figure S2**] has been characterized from the graphene/GaSe-nanosheet phototransistor by keeping *V*_SD_ =1 V and *V*_BG_=0 V. By fitting the curve using the 1/f noise equation *I*_n_^2^ µ 1/*f* ^β^ a factor *β*»1.13 can be derived, indicating it is 1/f like noise performance.


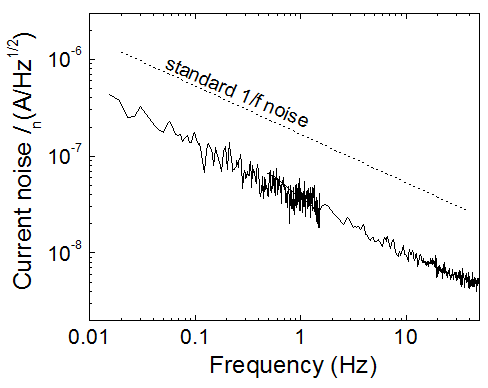


**Figure S2:** Noise spectrum of graphene/GaSe-nanosheet phototransistor in the case of *V*_SD_=1 V and *V*_BG_=0 V.

**References:**

1. V. Chikan, D. F. Kelley, *Nano Letters* **2002**, *2*, 141.

2. V. Chikan, D. F. Kelley, *Nano Letters* **2002**, *2*, 1015.

3. D. V. Rybkovskiy, A. V. Osadchy, E. D. Obraztsova, *J. Nanoelectron. Optoelectron.* **2013**, *8*, 110.

4. V. Grasso, Electronic Structure and Electronic Transitions in Layered Materials. Reidel Publishing Company: Dordrecht 1986.

5. P. A. Lee, Optical and Electronic Properties. D. Reidel: Dordrecht: 1976; Vol. 4.

6. J. M. Auerhammer, E. R. Eliel, *Optics Letters* **1996**, *21*, 773.

7. O. A. Balitskii, E. Borowiak-Palen, W. Konicki, *Crystal Research and Technology* **2011**, *46*, 417.

8. Y. K. Verma, R. H. Inman, C. G. L. Ferri, H. Mirafzal, S. N. Ghosh, D. F. Kelley, L. S. Hirst, S. Ghosh, W. C. Chin, *Physical Review B* **2010**, *82*, 165428.

9. H. Tu, S. Yang, V. Chikan, D. F. Kelley, *J. Phys. Chem. B* **2004**, *108*, 4701.

10. V. Chikan, D. F. Kelley, *Journal of Chemical Physics* **2002**, *117*, 8944.
